# Botanical Report

**Published:** 1811-09

**Authors:** 


### BOTANICAL REPORT.

The second volume of the Hortus Kewensis, which has been published since the ever-to-be-lamented death of Mr. Dryander, appears by by its contents to have undergone, throughout, the revision of that eminent botanist. We observe, however, in many parts, marks of haste; and doubts or difficult cases are frequently slurred over without that careful investigation and lucid determination which was to have been expected from his abilities, and which would, most certainly, not have been wanting, had he entered upon his task con amore.

Professor Wildenow's Species Plantarum is generally followed, and, as being the completest catalogue, and now in the hands of every botanist, there is certainly a considerable convenience in this; yet we cannot help regretting, that the references to Linnæus's own edition of the Species should not have been preserved, the more especially, as these have been so frequently omitted by Wildenow himself.

In the Asclepiadeæ, Mr. Brown's Genera, as published by him in the transactions of the Wernerian Society, are adopted, which makes a considerable change in the arrangement of the species belonging to that natural order. Something was necessary to be done, and perhaps the author could not do better than to follow the system of Mr. Brown, who has taken great pains with it, and made more observations upon this order than any other botanist. Linnæus had distinguished his genera by the form of what he called the nectaria, but many of the species unknown in his time, would not arrange under any of his genera, it became therefore necessary either to increase the number of genera, or to find different characters that would include such as had nectaria variously formed. If the difference had appertained to these organs only, the latter would have been the most proper plan. But an essential difference in the form of other parts of the plant being conjoined with the variation in this organ, nature seemed to point out a real generic distinction, and Mr. Brown has accordingly increased the number of genera, correspondent with the variety of forms in these essential parts. And we are satisfied that, in the end, this change of names, however much to be deprecated when made unnecessarily, will both promote the science and ease the labour of the student in his researches. We should have been glad, however, that means could have been found, of limiting, in some degree, the number of genera, in those cases especially where the species are not numerous. Several of Mr. Brown's genera consist of only one species as far as appears at least by this extensive catalogue. Thus Periploca is limited to two species, the *græca* and *lævigata*. *Indica* and *Secamone* are both separated into distinct genera, the latter under the name of the species, the former under that of Hemidesmus. Ceropegia, *sagittata* has the name of Microloma; Cynancum *viminale* that of Sarcostemma; *C. extensum* that of Dæmia; *C. erectum* that of Marsdenia. Asclepias *procera & gigantea* are raised into a genus under the name of Calotropis; A. *undulata* is called Xysmalobium; A. *carnosa* Hoya, after Mr. Hoy, gardener to the Duke of Northumberland, at Sion-house, an enthusiastic botanist, who, with proper encouragement, would do much for the promotion of the science. On the other hand Stapelia, an overgrown genus, containing four and forty species species, remains as it was, though many of the species are so different in the form of the nectarium, that they might as readily be separated into well defined genera as any of the natural order. But Mr. Brown does not seem to have had any thing to do with this genus; the Linnean character is preserved, and the term nectarium used, which Mr. Brown, in imitation of Jussieu and other French botanists has laid aside. The term he has used for this organ in the asclepiadeæ is *corona staminea*. In the rest of the class Pentandria there is little more of novelty; few species even that do not occur in Wildenow are recorded.

In the class *Hexandria* there is a greater accession of new matter, which chiefly arises from the attention Mr. Ker has paid to these plants, and the number that through him have been brought forward in the Botanical Magazine. But Mr. Dryander has, for the most part, arranged the species under the genera, in Wildenow's species plantarum, and has not paid the same regard to the observations of Mr. Ker, as he has done in the plants belonging to the natural order of ensatæ. This, we imagine, has arisen from a dislike on the part of Mr. Dryander, to take the trouble of framing new specific characters, which he must have done had he followed Mr. Ker, whose verbose descriptions could not be permitted to supply the place of Linnean definitions. Jacquin's genus Strumaria is adopted, and Wildenow's Hæmanthus *spiralis* referred to it; but, by an oversight, not Amaryllis *crispa*, which undoubtedly belongs to it, as observed by Ker. We observe that Narcissus *calathinus* of the Botanical Magazine is referred to *odorus*, and the *odorus* of the latter work is made another species for which Salisbury's name of *lætus* is adopted; so *trilobus* of Ker is said not to correspond with the description of Linnæus, and Haworth's name *nutans* is adapted for it; no *trilobus* however occurs in the genus.

Pancratium *rotatum* of Ker is preserved, and a new specific character applied to it, as also to P. *Amancaes* of the same, which Ruez and Pavon had referred to Narcissus, probably for no other reason, than its having yellow flowers. P. *caribæum* of Botanical Magazine is referred to *speciosum*.

Amaryllis *advena* of Ker, is admitted with a new character. A. *ornata* has likewise a new character, and the African and Ceylon plants considered with Ker as varieties, but A. *gigantea*, which the latter botanist had also considered as a variety, is excluded. We are inclined to think them all three distinct species. Brunsvigia of Heister is adopted from Ker, and A. *multiflora*, *marginata*, *radula* and *striata* referred to it.

Curculigo *orchioides* β of Ker is made a distinct species, and named *brevifolia*. Gethyllis plicata of Jacquin is referred to this genus, and two more new species are added. Aletris *fragrans* of Wildenow is referred to Dracæna after Ker; and the genus Tritoma of the latter author is adopted; so that the genus of Aletris is reduced to a single species, the *farniosa*. Smilacina is not separated from Convallaria, and Dracæna *borealis* of Wildenow, is retained; no reference, however, is made to the first edition of Aiton's Hortus Kewensis, where a figure of it was given; nor is even the distinction distinction of a variety given between this and the one figured in the Botanical Magazine, which Mr. Ker has since confessed to be a distinct species, and has applied Michaux's name of *umbellata* to it. Convallaria japonica of Wildenow is after Ker, separated under the name of Ophiopogon.

Ornithogalum *altissimum* is retained, though Ker without hesitation has referred it to Drimia.

In the genus Hyacinthus, Wildenow is exclusively followed; even Scilla nutans of Dr. Smith is retained, as Hyacinthus *non scriptis*; and H. *corymbosus* is not referred to Massonia with Ker. Scilla *romana* of Botanical Magazine remains with Hyacinthus; nor is Muscari separated. H. *serotinus* is with Wildenow referred to Lachenalia.

In Lachenalia *tricolor*, p. 288, a. 15. the trivial name is by accident omitted. L. *quadricolor* α and β of Botanical Magazine, are both referred to pendula, but we think they are more nearly allied to *tricolor*; indeed Mr. Ker has shewn that it is really the original *tricolor* of Hortus Kewensis.

In the genus Aloë Haworth's monograph in the Linnean transactions, is more especially followed in all the species which are not in Wildenow, but a different division of them into sections is followed, from the shape of the corolla only. We were rather surprised to find that the English name of *Cob-web* Aloe should be retained for A. Arachnoides, after Mr. Ker had pointed out that the name was derived from the similarity of the termination of the leaf to a spider's feet; and not, as in Sempervivum, from fine hairs spread over them like a web, of which there is not the smallest appearance in any of the varieties of this Aloë.

In the class Octandria occurs the immense genus erica, consisting of no fewer than one hundred and eighty-six species. Mr. Dryander appears to have taken more pains with this genus than any other in either volume of this work. He has arranged the whole under sections so well defined, that his specific characters, all of which are new, are beautifully concise and luminous. We consider it as the best example for illustrating an extensive genus, that is any where to be found. There is an inconvenience, however, attending the mode of printing the definitions of the sections, which being done in the same type, and in lines beginning parallel with the numbers of the species, are not easily caught by the eye. On this account we think we shall be rendering a service to our botanical readers by bringing the whole of the sections under one view, referring to the page in the work before us. We shall likewise translate them into English, with the intent of adding to the general utility of this synoptical table.

1.—*Macrostemones* (having large stamens). *Anthers* exserted, *i. e.* protruded beyond the corolla, and in all unarmed, *i. e.* having no appendix at the point of the filament. Page 360.

A. *Filaments* longer than the corolla, closely connivent (converging to a point), the part beyond the corolla of the same colour as the anthers. *Leaves* ternate (growing by threes). *Bractes* close to to the calyx (which Linnæus calls an imbricate calyx) in all except in E. Plukenetiana. *Limb of Corolla* erect, in all except E. Banksii. [This section contains Sp. 1— 8.]

B. *Filaments* nearly as long as the corolla (in E. umbellata, somewhat longer than corolla). *Flowers* terminal. *Leaves* ternat. *Flowers* ternate in all except E. bruniades and E. umbellata. [Sp. 9—18.]

C. *Anthers* exserted. *Flowers* axillary. *Leaves* linear in all except E. latifolia. *Bractes* remote from the calyx. *Limb of corolla* erect in all except in multiflora and grandiflora. Filaments erect in all except in staminea. [Sp. 19—26.]

2.—Longifloræ (Longflowered). *Corollas* cylindrical or club-shaped, exceeding half an inch in length. Page 368.

A. *Anthers* aristate (awned) *i. e.* having two linear or subulate appendages at the point of the filament, with an entire or a serulate margin. [Sp. 27—37.]

B. *Anthers* unarmed, *i. e.* having no appendages at the point of the filament. *Leaves* terminate. *Flowers* terminal. [Sp. 38—42.] Page 371.

C. *Anthers* unarmed. *Leaves* by fours (4—6 in E. concinna. 3—4 in E. flammea.) *Flowers* terminal; few varying from one to eight. [Sp. 43—52.]

D. *Anthers* unarmed. *Leaves* by fours. *Flowers* terminal, by fours, pressed closely into a square head. [Sp. 53—56.]

E. *Anthers* unarmed. *Leaves* by fours or more (frequently by sixes). *Flowers* axillary. *Bractes* close to the calyx. [Sp. 57—62.]

F. *Anthers* unarmed. *Leaves* by fours or more (frequently six.) *Flowers* axillary. *Bractes* two close to the calyx, and one distant. [Sp. 63—67.]

G. *Anthers* unarmed. *Leaves* by fours or more (frequently six). *Flowers* axillary. *Bractes* distant from the calyx. [Sp. 68—72.] Page 378.

3.—Conifloræ grandes (large cone-flowered). *Corollas* dilated downwards, exceeding half an inch in length. Page 380.

A. *Anthers* awned. [Sp. 73—78.]

B. *Anthers* unarmed. *Flowers* terminal. (In E. tetragona the flowering branches together with the flowers, being shorter than the leaves, the flowers appear to be axillary. [Sp. 79—91.]

4.—Calycinæ (having large calyxes. *Calyx* as long as the tube of the corolla, or of the whole corolla, or even longer than the corolla; coloured (not green) in all except in capitata, in which they are yellowish green. Page 385.

A. *Anthers* cristate or combed (*i. e.* having roundish or oblong appendages sawed at the edge.) *Leaves* by threes in all except in E. squamosa. [Sp. 92—99.]

B. *Anthers* armed. [Sp. 100—103.]

C. *Anthers* unarmed. [Sp. 104—111.]

5.—Brevifloræ (short flowered). *Corollas* exceeding a quarter but not more than half an inch long: *Tube* longer than the calyx. Page 390.

A. *Tube*

A. *Tube of Corolla* nearly globular. *Anthers* cristate in all except in E. odorata. [Sp. 112—118.]

B. *Tube of Corolla* urseolate (pitcher shaped.) *Flowers* axillary. *Bractes* close to the calyx. [Sp. 119—121.]

C. *Corollas* dilated downwards (conical or ovate) or oblong and contracted at the mouth. [Sp. 122—133.]

D. Corollas cylindrical or dilated upwards. [Sp. 134—144.]

6.—PARVIFLORÆ (small-flowered.) *Corollas* not exceeding a quarter of an inch in length: *Tube* longer than calyx. Page 398.

A. *Anthers* cristate. *Calyx* erect in all except in E. Bergiana. [Sp. 145—155.]

B. *Anthers* awned. *Leaves* by threes. [Sp. 156—164.]

C. *Anthers* awned. *Leaves* by fours or more. [Sp. 165—174.]

D. *Anthers* unarmed. *Leaves* linear in all except in E. cordata and hispidula. [Sp. 175—186.]

This division, although artificial, brings together for the most part such as appear by their habit to be nearly allied. It is not however to be supposed but that some new species may be discovered, which will not very readily arrange under any of these divisions. Some inconvenience will, we doubt not, be felt by a reference to a positive measurement of the corolla; as some species may vary, so as sometimes to be below and sometimes to exceed half an inch. We regard this as the greatest defect in this arrangement, which is nevertheless most excellent.

